# miR-195-3p/BDNF axis regulates hypoxic injury by targeting P-ERK1/2 expression

**DOI:** 10.1097/MD.0000000000031586

**Published:** 2022-11-18

**Authors:** Wenjing Zhang, Bingshi Liu, Yanfang Wang, Lixian Sun PHD, Chao Liu, Haoran Zhang, Wei Qin, Jingyi Liu, Leng Han, Weichao Shan

**Affiliations:** a Department of Cardiology, Affiliated Hospital of Chengde Medical University, Chengde, China; b Department of Cardiology, Pingquan City Hospital, Chengde, China.

**Keywords:** apoptosis, atherosclerosis, brain-derived neurotrophic factor, human umbilical vein endothelial cell, hypoxia injury, miR-195-3p, proliferation

## Abstract

Coronary heart disease (CHD) is the most common heart disease and the leading cause of cardiovascular deaths worldwide. Decreased endothelial cell (EC) proliferation, increased apoptosis, inflammation, and vascular dysfunction are considered vital factors in CHD. In this study, we aimed to determine the expression and role of microRNA-195-3p and brain-derived neurotrophic factor (BDNF) in hypoxic-treated human umbilical vein endothelial cells (HUVECs).

**Measures:**

We induced hypoxia in HUVECs using the “anaerobic tank method.”

**Results:**

We found that the levels of microRNA-195-3p and BDNF were upregulated and apoptosis was increased. Furthermore, we found that BDNF/P-ERK1/2 regulated the expression of the mitochondrial apoptosis pathway proteins Bcl-2/BAX, which was downregulated under hypoxic conditions. Finally, the microRNA-195-3p inhibitor downregulated BDNF and P-ERK1/2, upregulated the Bcl-2/BAX axis, and partially reversed the effects of hypoxic-induced injury in HUVECs.

**Conclusions:**

Therapeutic intervention using the microRNA-195-3p/BDNF/P-ERK1/2/Bcl-2/BAX axis could maintain EC function under hypoxic conditions, improve cell activity, and serve as a new treatment strategy for CHDs.

## 1. Introduction

Coronary heart disease (CHD) and its cardiovascular sequelae are leading causes of mortality worldwide.^[1]^ Atherosclerotic plaques in the coronary arteries form a thrombus, leading to stenosis or even blockage of the vascular cavity, eventually causing ischemia, hypoxia, and necrosis of myocardial cells. Endothelial cells (ECs) are key players in the progression and regression of atherosclerotic disease. Despite significant advances in revascularization and intensive medical care interventions, patients with CHD are associated with a persistently high rate of myocardial infarction and death.^[1]^ Owing to the unsatisfactory outcomes of current treatments, the development of novel therapeutic strategies is needed.

microRNAs (miRNAs) are small endogenous noncoding RNAs that modulate gene expression either by inhibiting the translation or by promoting the degradation of messenger RNA (mRNA). The regulatory mechanisms of miRNAs include cell differentiation, proliferation, apoptosis, and tumorigenesis.^[2]^ Recent studies have shown that miR-195 plays a critical role in many cardiovascular diseases,^[3]^ such as cardiac hypertrophy/heart failure, interstitial fibrosis, and diabetic cardiomyopathy.^[4–6]^ miR-195 − 3p and miR-195 − 5p are the 2 mature forms of miR-195.^[7]^ Although several studies have revealed that miR-195-3p is associated with several cardiovascular diseases, its role in hypoxia-induced apoptosis in ECs and the underlying mechanisms are not fully understood.

Brain-derived neurotrophic factor (BDNF) is an immediate upstream regulator of ERK1/2,^[8]^ which play key regulatory roles in neuronal activity and cognitive functions. Recently, BDNF has been shown to promote neovascularization of ischemic tissue by recruiting ECs.^[9]^ Human umbilical vein endothelial cells (HUVECs) are primary ECs involved in angiogenesis and vasculature formation.^[10]^ In this study, we investigated the role of miR-195-3p in the regulation of hypoxic injury in HUVECs. However, little information is available regarding the molecular mechanisms underlying these effects. Our study showed that miR-195-3p binds to the 3′-untranslated region (3′-UTR) of BDNF. We further mechanistically determined the effects of the BDNF/ERK1/2/Bcl-2 axis on the proliferation and apoptosis of HUVECs. In addition, the miR-195-3p inhibitor reversed hypoxia for 6 hours by targeting the BDNF protein. Therefore, our results revealed for the first time that the miR-195-3p/BDNF/ERK1/2/Bcl-2/Bax axis is involved in hypoxia in HUVECs. These findings provide a new therapeutic strategy for EC survival and microvessel remodeling.

## 2. Materials and Methods

### 2.1. Patient and public involvement

No patient involved.

### 2.2. Cell culture

HUVECs (Beijing Being Chuanglian Biotechnology Institute, Beijing, China) were cultured in Dulbecco’s modified Eagle’s medium (DMEM, Gibco) supplemented with 1% penicillin and streptomycin (Solarbio, Beijing, China) and 10% fetal bovine serum (FBS, Gibco, Shanghai, China) at 37°C and 5% CO_2_. Cells in the logarithmic phase were collected for use in subsequent experiments.

### 2.3. Induction of hypoxia

HUVECs were treated according to the “anaerobic tank method”^[11]^ to develop hypoxia-induced EC injury in vitro. Briefly, HUVECS were cultured in low-glucose DMEM without FBS at 37°C in an incubator maintained at 95% N_2_ and 5% CO_2_ for 6 hours. Following the successful establishment of the hypoxic model, cells were used for subsequent experiments.

### 2.4. Western blotting

The levels of protein expression were analyzed using western blotting. Total protein was quantified, and 30 µg of protein per lane was separated using 8% sodium dodecyl sulfate polyacrylamide gel electrophoresis and transferred to polyvinylidene fluoride (PVDF, Roche, Shanghai, China) membranes. Membranes were then incubated with the following primary antibodies at 4°C for 6 hour: GAPDH (1:6000, no. A19056), vinculin (1:1000, no.A2752) (ABclonal, Wuhan, China), BDNF (1:5000, no. #ab108319) (Abcam, Cambridge, UK), caspase-3 (1:1000, no. #14220), cleaved caspase-3 (1:1000, no. #9664), cytochrome C (1:1000, no. #11940), hypoxia-inducible factor 1 subunit alpha (HIF1A; 1:1000, no. #14179), Bax (1:1000, no. #2772), Erk1/2 (1:1000, no. #4696), P-ERK1/2 (1:1000, no. #4370), and Bcl-2 (1:1000, no. #4223) (Cell Signaling Technology, Danvers). Following incubation with primary antibodies, membranes were washed and incubated with secondary antibodies (goat anti-rabbit IgG, ab182016; Abcam) at 25°C for 2 hour. Protein bands were visualized using an enhanced chemiluminescence reagent (Wanleibio, Shenyang, China). Bands were quantified using the ImageJ software. GAPDH or vinculin was used as an internal control.

### 2.5. Bioinformatics analysis

TargetScan (http://www.targetscan.org/vert_72/) was used to predict target genes of miR-195-3p. In total, 869 target genes were identified. BDNF was searched to obtain the BDNF 3′-UTR containing the miR-195-3p binding sites.

### 2.6. Cell transfection

HUVECs were seeded at a density of 6 × 10^4^/cm^2^ in a plate and allowed to settle for 24 hours to ensure 80% to 90% confluence before transfection.

Transfection sequence (5′ to 3′)(Sangon Biotech, Shanghai, China) was performed as follows: inhibitor NC: CAG UAC UUU UGU GUA CAA, inhibitor miR-195-3p:GGAGCAGCACAGCCAAUAUUGG; mimics: N.C sense: UUGUACUACACAAAAGUACUG, N.C antisense: GUACUUUUGUGUAGUACAAUU, miR-195-3p sense: CCAAUAUUGGCUGUGCUGCUCC, miR-195-3p antisense: AGCAGCACAGCCAAUAUUGGUU.

HUVECs were transfected with a mixture containing 10 μL (50 nM) inhibitor, 10 μL lipofectamine 3000 (Invitrogen, Thermo Fisher Scientific, Shanghai, China), and 980 μL Opti-MEM (Gibco) in DMEM supplemented with 1% FBS. Cells transfected with negative control (NC) inhibitors were used as controls. After 48 hours, transfected cells were subjected to hypoxic conditions for 6 hours. Transfection efficiency was determined by RT-qPCR.

### 2.7. Qrt-qPCR

The DNA/RNA isolation kit (Tiangen, Beijing, China) was used to extract total RNA. Primers were designed and synthesized by TIANGEN BIOTECH Co., LTD). The following primers were used: U6 forward, 5′-CTGGCTTCGGCAGCACA-3′; reverse, 5′-AACGCTTCACGAATTTGCGT-3′. has-miR-195-3p primer sequence: forward, 5′-CCAAUAUUGGCUGUGCUGCUCC-3′. RNA was reverse transcribed into cDNA using a cDNA reverse transcription kit (Tiangen) following the manufacturer’s instructions. The following primes were used: BDNF (GeneCopoeia, Guangzhou, China) forward, 5′-GGCTTGACATCATTGGCTGAC-3′, reverse, 5′-GCCGAACTTTCTGGTCCTCAT-3′; GAPDH forward, 5′-GCACCGTCAAGGCTGAGAAC-3′, reverse, 5′-TGGTGAAGACGCCAGTGGA-3′. The RT-qPCR reaction system (20 µL) contained 2 µL cDNA, according to the manufacturer’s instructions. U6 or GAPDH served as internal controls. The relative transcription levels of target genes were calculated using the 2−ΔΔCq method. ΔΔCq was calculated using the following equation: ΔΔCq = [Cq (target gene) − Cq (internal reference gene)] experimental group − [Cq (target gene) − Cq (internal reference gene)] control group.

### 2.8. 5-ethynyl-2′-deoxyuridine assay

Cells were seeded in 48-well plates for 24 hours and incubated in medium containing 5-ethynyl-2-deoxyuridine (EdU) (50 mM; RiboBio Co., Guangzhou,China) for 2 hours. Cell fixation, staining, and DNA staining were performed according to the manufacturer’s instructions. Nuclei and EdU-positive cells were observed under a fluorescence microscope (Nikon, Tokyo, Japan), and the proliferation rate (EdU-positive cells/nucleus) was calculated in random fields.

### 2.9. Cell counting kit-8 assay

The viability of HUVECs was assessed using the cell counting kit-8 (CCK-8, Dojindo, Japan) assay, as previously described. In brief, cell density was 1 × 10^3^ cells/well. After treatment, 90 mL DMEM containing 10 mL CCK-8 solution was added to each well, and cells were incubated for 2 hours at 37°C. Absorbance was determined using a spectrophotometer at 450 nm (BioTek, Winooski, VT).

### 2.10. Terminal deoxynucleotidyl transferase dUTP nick-end labeling assay

HUVECs were cultured in a 24-well plate, transfected with 50 nmol/L miR-195-3p inhibitor or NC inhibitor (control), and subjected to hypoxia for 6 hours. Cells were stained using the terminal deoxynucleotidyl transferase dUTP nick-end labeling (TUNEL) apoptosis assay kit, (Roche, Basel, Switzerland), and then viewed under a fluorescence microscope (OlympusCorporation, Japan).

### 2.11. Statistical analysis

Data were analyzed using GraphPad Prism software 8.0. Data are presented as the mean ± standard deviation. A 2-sided Student *t* test was used to analyze individual differences, and statistical significance was set at *P* < .05.

## 3. Results

### 3.1. Establishment of in vitro hypoxia-induced model

**Figure 1.**
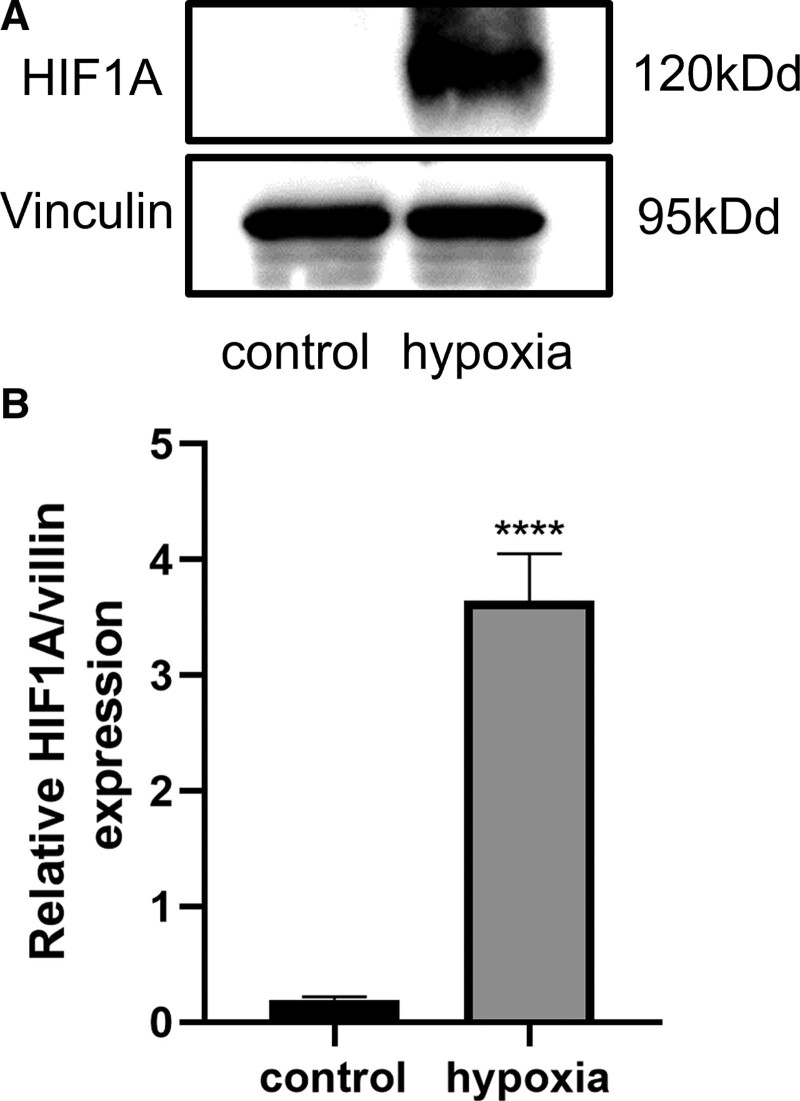
In vitro hypoxic model in HUVECs. (A) The levels of expression of the hypoxia-related HIF1A protein were increased after induction of hypoxia for 6 h. (B) Quantification of the levels of expression of HIF1A after induction of hypoxia for 6 h. All values are the mean of 3 independent experiments (n = 3). ****P < .0001 versus control. HIF1A = hypoxia inducible factor 1 subunit alpha, HUVECs = human umbilical vein endothelial cells.

We detected the level of expression of hypoxia-inducible factor 1-alpha (HIF1A) in HUVECs using western blotting. We specifically found that compared with the control group, the level of expression of HIF1A was significantly (*P* < .0001) increased in hypoxia-induced HUVECs (Fig. 1A and B), suggesting the successful establishment of an in vitro hypoxia-induced model.

### 3.2. Regulation of mir-195-3p and BDNF in response to hypoxia

**Figure 2.**
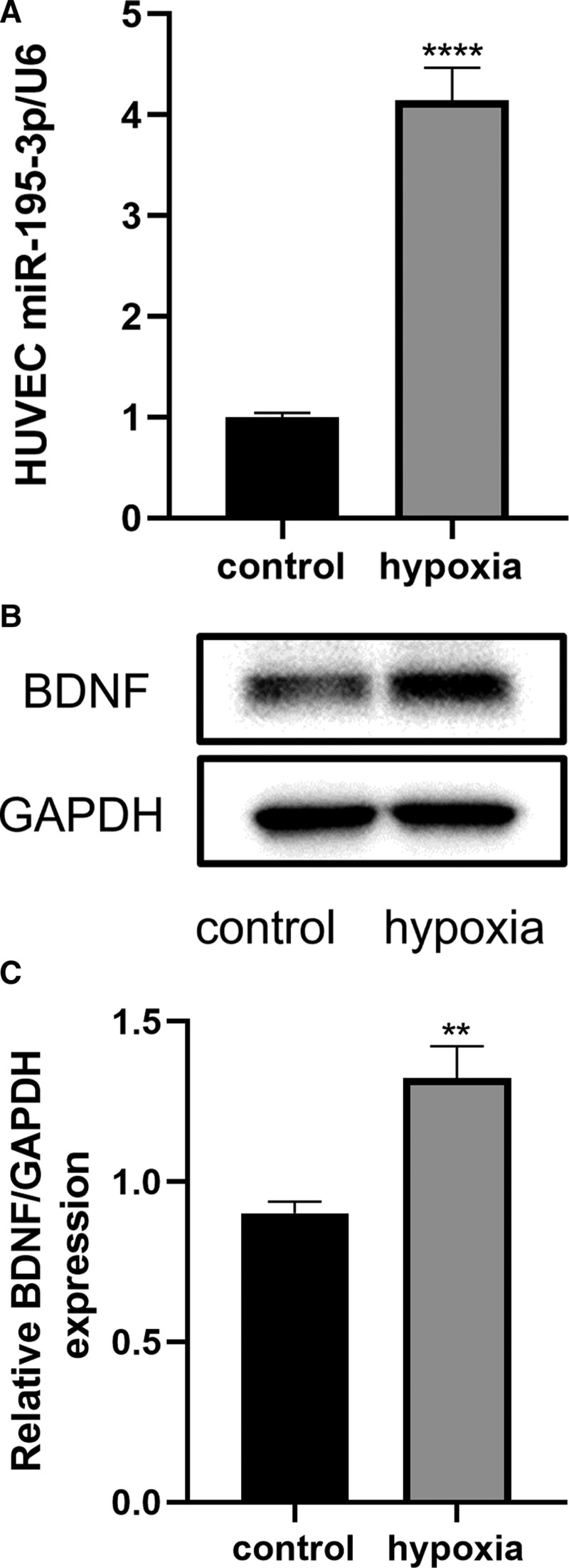
miR-195-3p and BDNF are involved in the process induced by hypoxia in HUVECs. (A) The levels of expression of the miR-195-3p-related BDNF protein were increased after induction of hypoxia for 6 h compared with those in the control group. (B and C) Quantification of the levels of expression of BDNF after induction of hypoxia for 6 h. All values are the mean of 3 independent experiments (n = 3). **P < .01, ****P < .0001 versus control. BDNF = brain-derived neurotrophic factor, HUVECs = human umbilical vein endothelial cells, miR-195-3p = microRNA-195-3p.

We evaluated the expression of miR-195-3p in hypoxia-induced HUVECs. We observed that the expression of the miR-195-3p gene was significantly increased (*P* < .0001) (Fig. 2A), and at the same time, the expression of BDNF was increased after 6 hours of hypoxia treatment (Fig. 2B and C) compared with the control group (*P* < .01). This finding suggested that miR-195-3p and BDNF play a role in hypoxic injury in HUVECs.

### 3.3. Transfection with mir-195-3p mimic and inhibitor

**Figure 3.**
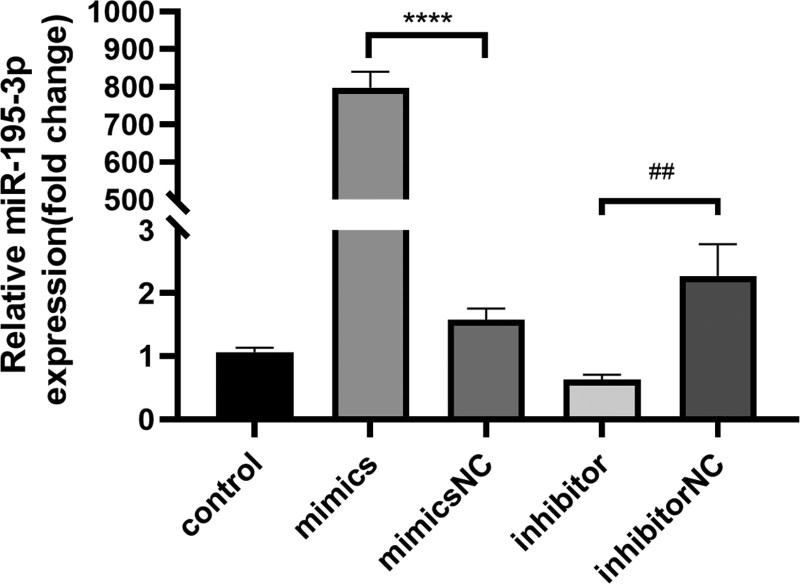
The levels of expression of miR-195-3p in different treatment groups. HUVECs were transfected with miR‑195-3p mimic, NC mimic inhibitor, and NC inhibitor. After 48 h the levels of expression of the miR-195-3p gene were measured by RT-qPCR. All values are the mean of 3 independent experiments (n = 3). ****P < .0001 versus mimic group, ##*P* < .01 versus NC mimic group. HUVECs = human umbilical vein endothelial cells, miR-195-3p = microRNA-195-3p, NC = negative control.

To clarify whether miR-195-3p is involved in hypoxic injury, we performed liposome-mediated transfection of HUVECs with the miR-195-3p inhibitor or mimic. We further validated the level of expression of miR-195-3p following transfection with the mimic, using real-time PCR. We accordingly detected that the gene expression of miR-195-3p was significantly (*P* < .0001) increased compared with that in cells transfected with the mimic NC. Conversely, we noticed that compared with the inhibitor NC, transfection with the miR-195-3p inhibitor reduced (*P*<.01) the gene expression of miR-195-3p (Fig. 3). These findings strongly supported that the miR-195-3p mimic and inhibitor were successfully transfected into HUVECs, resulting in the upregulation and downregulation of miR-195-3p, respectively.

### 3.4. Mir-195-3p regulated the expression of BDNF by directly binding to the 3′-UTR of BDNF

**Figure 4.**
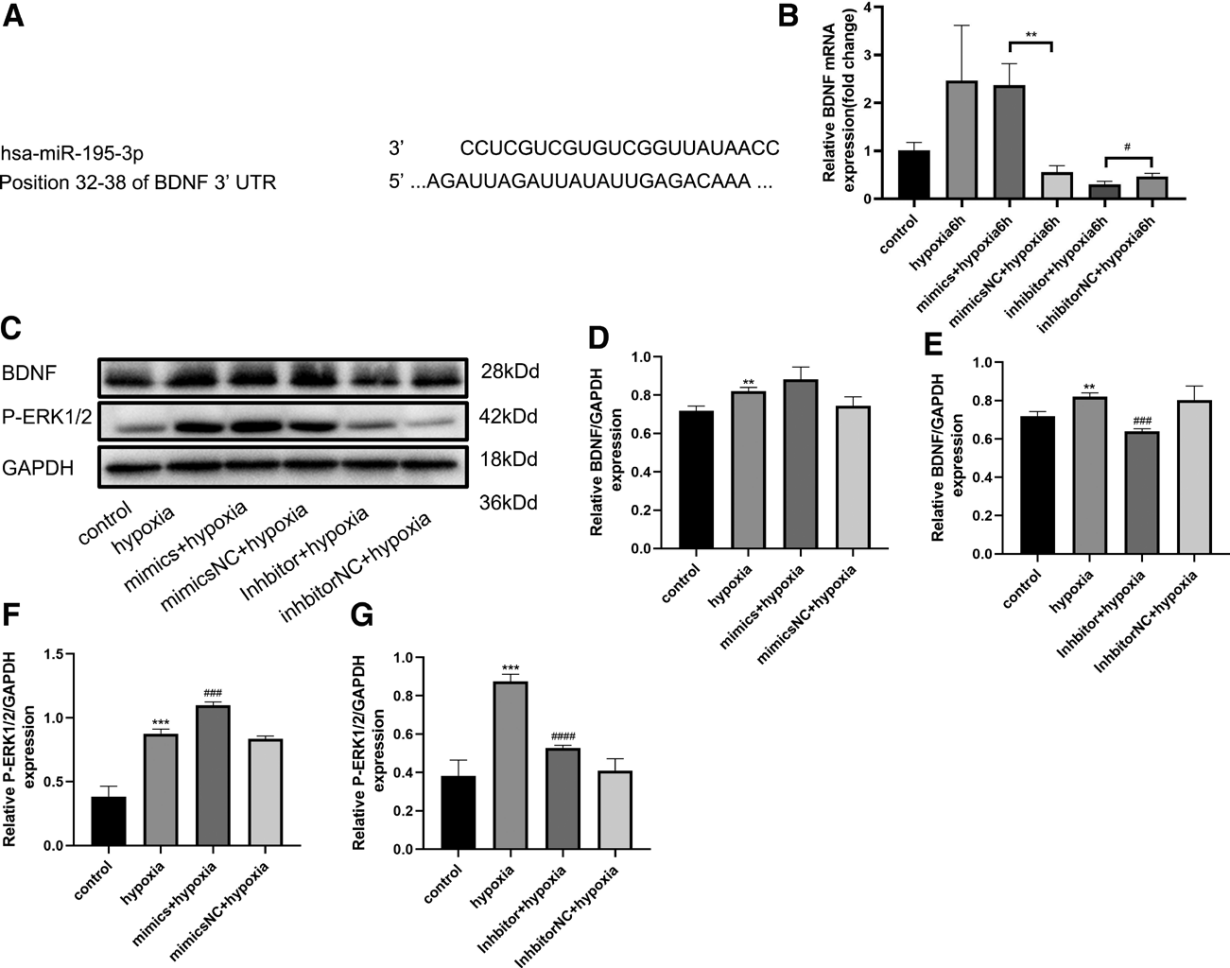
BDNF is a direct target of miR-195-3p after hypoxia for 6 h. (A) Schematic of the BDNF 3′-UTR containing the miR-195-3p binding sites. (B) HUVECs were transfected with miR-195-3p mimic, inhibitor, and NC mimic separately. HUVECs were subjected to hypoxia for the indicated time. The relative level of expression of BDNF was detected by RT-qPCR. All values are the mean of 3 independent experiments (n = 3). (C–G) HUVECs were transfected with miR-195-3p mimic, NC mimic inhibitor, and NC inhibitor. After 48 h, cells were subjected to hypoxia for 6 h. The levels of expression of BDNF and P-ERK1/2 were determined by western blotting. GAPDH served as an internal control. **P < .01, ***P < .001 versus control group. ###*P* < .001, ####*P* < .0001 versus hypoxic group. BDNF = brain-derived neurotrophic factor, HUVECs = human umbilical vein endothelial cells, miR-195-3p = microRNA-195-3p, NC = negative control.

Next, we investigated the downstream targets of miR-195-3p in the pathogenesis of hypoxic injury. TargetScan prediction revealed a binding sequence between miR-195-3p and the BDNF-mRNA 3′-UTR region (Fig. 4A). In addition, real-time PCR showed that after hypoxic exposure, the autocrine levels of BDNF were increased to a certain extent. Moreover, compared with the mimic NC group, we found that the mRNA expression of BDNF was significantly (*P* < .01) improved by the miR-195-3p-mimic in HUVECs, whereas it was significantly (*P* < .05) inhibited by the miR-195-3p-inhibitor (Fig. 4B). These results confirmed the binding between miR-195-3p and the BDNF-mRNA 3′-UTR region, and the upregulation of the mRNA expression of BDNF following hypoxia. Furthermore, we determined the levels of expression of BDNF and P-ERK1/2 using western blotting. We observed that the miR-195-3p-mimic significantly (*P* < .05) enhanced the expression of BDNF; concurrently, the expression of P-ERK1/2 was also enhanced. However, this process was alleviated by treatment with the miR-195-3p-inhibitor (Fig. 4C–G). These results suggested that miR-195-3p positively regulates the expression of the BDNF/P-ERK1/2 axis.

### 3.5. Mir-195-3p inhibitor rescued the apoptosis of hypoxia-induced injured HUVECs

**Figure 5.**
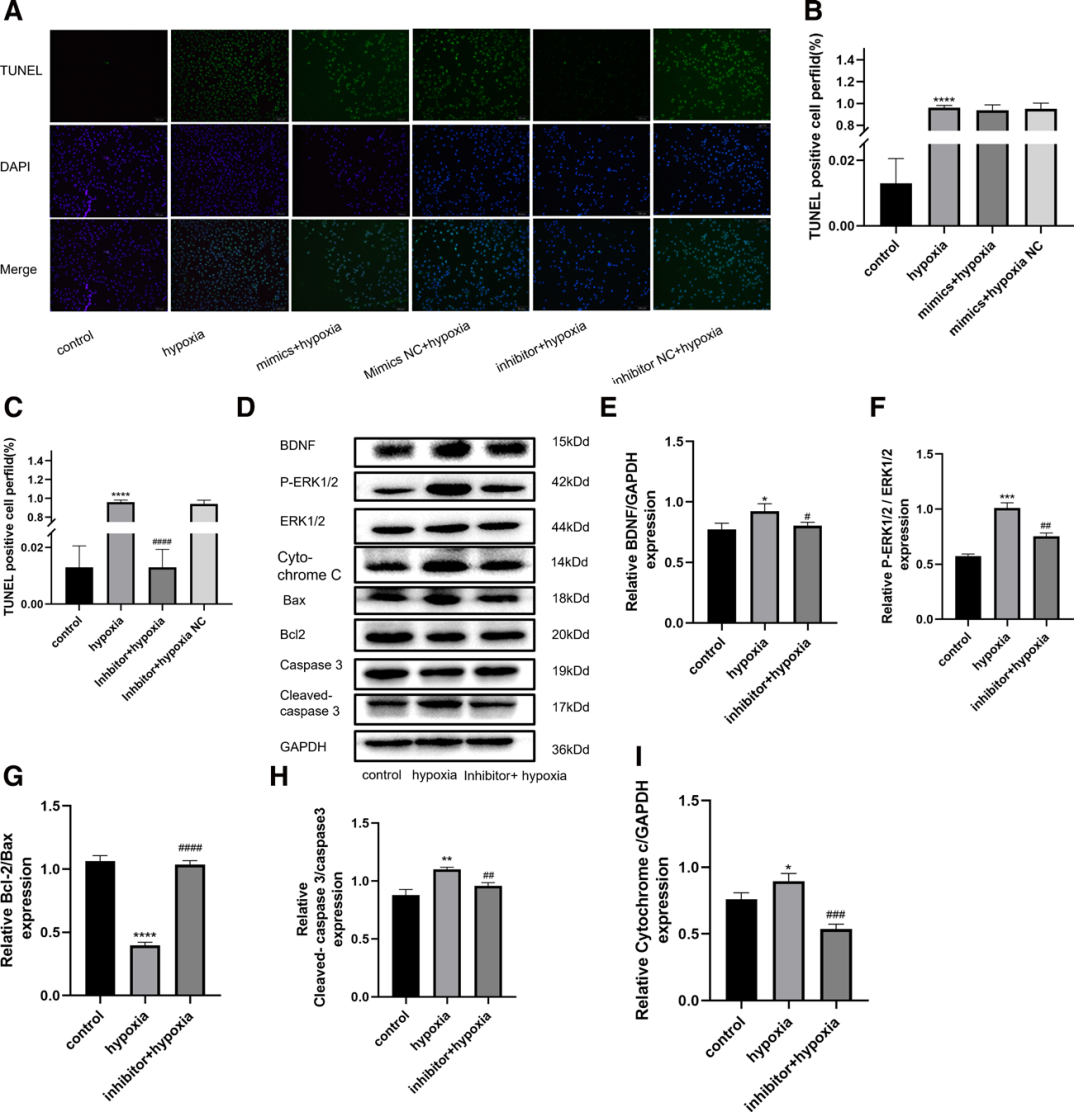
BDNF might partially attenuate hypoxia-induced HUVEC apoptosis by targeting miR-195-3p. (A) Representative images of TUNEL staining of HUVECs showing apoptotic cells (apoptotic cells stained in green and nuclei stained in blue with DAPI) (magnification, ×200). (B and C) Statistical analysis of TUNEL-positive cells per field (n = 3). (C) Western blotting of cells in the following groups: control, hypoxia induction for 6 h, and hypoxia induction (6 h) + miR-195-3p inhibitor. (D–I) Ratio of levels of expression of BDNF/GAPDH, P-ERK1/2 to ERK1/2, cleaved caspase-3/caspase-3, Bcl-2/Bax, and levels of cytochrome C. Bands were quantified using the Image J software. Each bar represents the mean ± standard deviation of 3 independent experiments. *P < .05, **P < .01, ***P < .001, ****P < .0001 versus the control group. #*P* < .05, ##*P* < .01, ###*P* < .001, ####*P* < .0001 versus hypoxia induction for 6 h group. DAPI = 4′,6-diamidino-2-phenylindole, HUVECs = human umbilical vein endothelial cells, TUNEL = terminal deoxynucleotidyl transferase dUTP nick-end labeling.

We also investigated the mechanisms underlying the effects of the BDNF/P-ERK axis on hypoxia-induced HUVEC apoptosis. After transfecting HUVECs with a miR-195-3p mimic and miR-195-3p inhibitor, we found that the expression of miR-195-3p in HUVECs was increased and decreased, respectively. We then subjected HUVECs to hypoxia to simulate the ischemic status in vitro. In addition, we performed TUNEL assay to delineate the role of miR-195-3p in hypoxia-induced cardiomyocyte apoptosis. We found that the number of TUNEL-positive cells was significantly (*P* < .0001) increased in the hypoxic group. Interestingly, this number was diminished by treatment with the miR-195-3p inhibitor but not by the miR-195-3p-mimic and miR-NC (Fig. 5A–C). These data implied that the miR-195-3p inhibitor prevented hypoxia-induced apoptosis of HUVECs. To verify whether the miR-195-3p inhibitor indeed inhibits apoptosis, we transfected HUVECs with the miR-195-3p inhibitor for 48 hours and then subjected them to hypoxia for 6 hours. We observed that in the hypoxia-induced group, the levels of expression of BDNF and ratio of P-ERK1/2 to ERK1/2 were increased, whereas the ratios of Bcl-2/Bax, cleaved caspase-3/caspase-3, and levels of cytochrome C were decreased compared with those in the control group. However, compared with the hypoxic group, the levels of expression of BDNF and ratio of P-ERK1/2 to ERK1/2 were decreased, whereas the ratios of Bcl-2/Bax, cleaved caspase-3/caspase-3, and levels of cytochrome C were increased after transfection with the miR-195-3p inhibitor (Fig. 5D–I). These results suggested that the miR-195-3p inhibitor-targeted BDNF/P-ERK1/2/Bcl-2/BAX axis reduced hypoxia-induced apoptosis of HUVECs.

### 3.6. Mir-195-3p inhibitor partially restored the proliferation of damaged HUVECs

**Figure 6.**
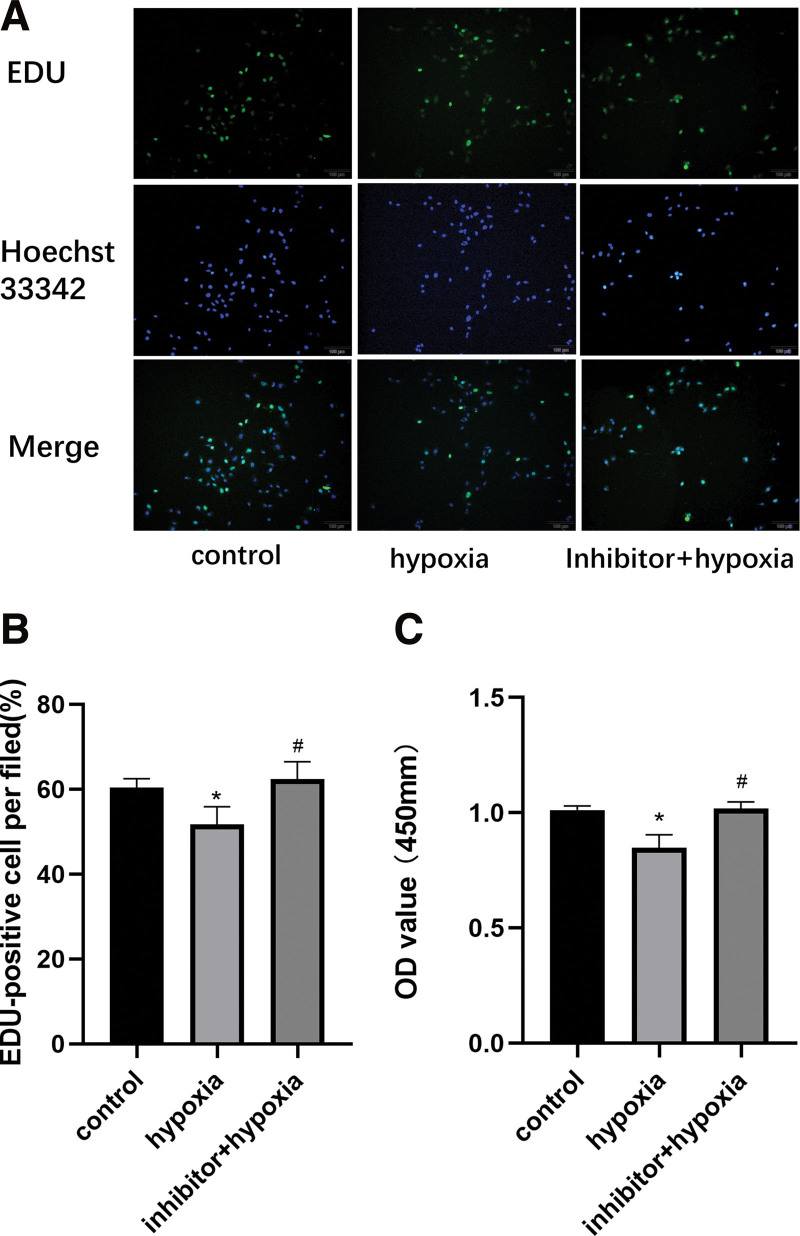
miR-195-3P inhibitor restores the proliferation rate of hypoxia-induced HUVECs. (A and B) EdU analysis shows the proliferation rate of each group. Cells were stained with Hoechst 33342 (5 μg/mL), and the proliferating population was analyzed (n = 3). Magnification, ×200. (C) The viability of cells in different treatment groups was measured using the CCK-8 assay, OD value = 450 mm, (n = 5). **P < .01, ***P < .001 versus the control group. ##*P* < .01, ###*P* < .001 versus hypoxia induction for 6 h group. CCK-8 = cell counting kit-8, EdU = 5-ethynyl-2′-deoxyuridine, HUVECs = human umbilical vein endothelial cells.

Next, we transfected HUVECs with the miR-195-3p inhibitor and induced hypoxia for 6 hours. Subsequently, we analyzed the proliferation rate and viability of cells. We observed that treatment with the inhibitor following hypoxia restored the number of EdU-positive cells compared with those in the control group (Fig. 6A and B). Similarly, the CCK-8 assay showed that transfection with the miR-195-3p inhibitor improved the viability of hypoxic-injured HUVECs, confirming a partial restoration of cell proliferation (Fig. 6C). In summary, the miR-195-3p inhibitor reversed the hypoxia-induced inhibition of cell proliferation.

## 4. Discussion

The present study demonstrated that miR-195-3p and BDNF were upregulated in hypoxia-induced HUVECs, increasing their apoptosis. Conversely, downregulation of miR-195-3p reduced hypoxic-induced apoptosis, increasing cellular viability. The miR-195-3p inhibitor mitigated the proapoptotic effects of the BDNF/P-ERK1/2 axis in HUVECs. These findings suggested that downregulation of the miR-195-3p/ BDNF/P-ERK1/2 axis might be beneficial for repairing the hypoxic injury in ECs.

Previous studies have shown that hypoxia induces vascular endothelial injury in HUVECs. HUVECs are primary EC involved in angiogenesis and vasculature formation and are related to signaling molecules and endothelial biomarkers controlling vascular homeostasis.^[12–15]^ The “anaerobic tank method” was used to establish the hypoxic model. The expression of HIF-1a in HUVECs^[16,17]^ was analyzed to confirm the successful establishment of an in vitro hypoxic model. In CHD, the accumulation of senescent ECs in the vascular lesion area is regarded as a vital factor contributing to vascular dysfunction.^[18–20]^ The senescence of ECs has been associated with increased apoptosis, decreased EC proliferation, and inflammation, all of which are involved in vascular dysfunction and disease.^[21–23]^ Similar observations were made in this study, in which the number of TUNEL-positive cells was increased after hypoxic injury in HUVECs. A decrease in the Bcl-2/Bax ratio and an increase in the levels of miR-195-3p, the cleaved caspase-3/caspase-3 ratio, and levels of cytochrome c confirmed the hypoxia-induced apoptosis of HUVECs.

miRNAs regulate gene expression by binding to the 3′-UTR of the target mRNA, resulting in translational repression.^[24,25]^ We focused on miR-195-3p, as this miR has been previously reported as a miR with potential proapoptotic properties and demonstrated for the first time the critical role of miR-195-3p in hypoxic-induced apoptosis and cellular function. In previous studies, astragalus polysaccharides were shown to suppress the proliferation and invasion of tumor cells by upregulating miR-195-5p in nonsmall cell lung cancer^[26]^ miR-195 has also been reported to inhibit the proliferation and enhance apoptosis of OSCC cells by targeting TLR4,^[27]^ while miR-195-5p was shown to inhibit the viability and proliferation and promote apoptosis of HUVECs.^[28–30]^ However, to the best of our knowledge, the effects of miR-195-3p on hypoxia-induced HUVECs have rarely been reported. Further treatment with an miR-195-3p inhibitor successfully downregulated its expression in HUVECs, where the number of TUNEL-positive cells was decreased after the induction of hypoxia. Mitochondrial proteins are known to directly regulate apoptosis. More specifically, Bax and Bcl-2 have been both implicated in caspase-associated apoptosis, and an increase in the Bax/Bcl-2 ratio is known to trigger apoptotic events, including the activation of caspase3/9 and subsequent degradation of intracellular substrates.^[31–33]^ Accordingly, the observed increase in the Bcl-2/Bax ratio, cleaved caspase-3/caspase-3 ratio, and levels of cytochrome C in our study confirmed that the knockdown of miR-195-3p alleviated apoptosis of HUVECs.

Whether BDNF is a direct target of miR-195-3p is controversial. To further explore the underlying molecular mechanism and predict the potential targets of miR-195-3p in cells, we performed bioinformatics analysis using TargetScan and found that the 3′-UTR of BDNF contained potential binding sites complementary to miR-195-3p. We validated the expression of BDNF after transfection with an miR-195-3p mimic or inhibitor by performing both qRT-PCR and western blot analyses. We found that after hypoxic exposure, the autocrine levels of BDNF were increased to a certain extent, indicating that the damage stimulated a repair feedback mechanism in HUVECs. In turn, the expression of p-Erk1/2 was increased, whereas the cell survival rate was decreased. As such, we concluded that this feedback mechanism was insufficient to completely repair the damage caused by hypoxic exposure. Then, we examined the effect of miR-195-3p on the expression of BDNF and found that the levels of BDNF were regulated by miR-195-3p. When miR-195-3p was upregulated, the expression of both the BDNF gene and protein was increased. In contrast, when miR-195-3p was downregulated, the expression of both the BDNF gene and protein was decreased. Therefore, our findings suggested that BDNF is a direct target of miR-195-3p. In the next step, we transfected HUVECs with a miR-195-3p inhibitor. Compared with the hypoxic-induced group, the level of expression of BDNF and ratio of PERK1/2 to ERK1/2 declined, whereas those of components of the mitochondrial apoptotic pathway, such as the ratios of Bcl-2/Bax, cleaved caspase-3/caspase-3, and levels of cytochrome C were increased. Conclusively, we speculated the protective role of the downregulation of the miR-195-3p/BDNF/P-ERK1/2/Bcl-2/BAX axis^[8,34]^ against ischemic apoptosis. Moreover, EdU and CCK-8 assays suggested that treatment with an inhibitor improved the proliferation of HUVECs following hypoxic injury.

## 5. Strengths and limitations of this study

This study was performed to investigate the expression and role of microRNA-195-3p (miR-195-3p) in the regulation of hypoxic injury in HUVECs. We believe that our study makes a significant contribution to the literature because we show the role of miR-195-3p and BDNF in hypoxic injury in HUVECs. Our study had several limitations. We only explored the function of ECs in vitro and did not verify these changes in vivo.

## 6. Conclusion

In this study, we demonstrated the role of BDNF and miR-195-3p in hypoxic injury in HUVECs. Treatment with an miR-195-3p inhibitor regulated the BDNF/P-ERK1/2/Bcl-2/BAX axis and partially reversed hypoxic injury in HUVECs, reduced apoptosis, and improved proliferation. Maintaining the normal function of ECs might provide new therapeutic strategies for treating CHD.

## Author contributions

WZ and WS performed the experiments, revised the manuscript and integrated the figures. YW and LS performed the experiments, and produced the original draft of the manuscript. CL and HZ helped with the cell biology experiments. WQ, JL, and LH helped with the experiments of molecular biology. BL also helped with the cell biology and molecular biology experiments, and analyzed the data. All authors contributed to and approved the final manuscript.

**Conceptualization:** Wenjing Zhang, Lixian Sun, Haoran Zhang, Wei Qin, Weichao Shan.

**Data curation:** Bingshi Liu, Wei Qin, Weichao Shan.

**Formal analysis:** Wenjing Zhang, Bingshi Liu, Wei Qin, Weichao Shan.

**Funding acquisition:** Wenjing Zhang, Jingyi Liu, Weichao Shan.

**Investigation:** Chao Liu, Jingyi Liu, Weichao Shan.

**Methodology:** Bingshi Liu, Chao Liu, Jingyi Liu, Weichao Shan.

**Project administration:** Chao Liu, Haoran Zhang, Jingyi Liu.

**Resources:** Lixian Sun, Leng Han, Weichao Shan.

**Software:** Yanfang Wang.

**Validation:** Yanfang Wang, Lixian Sun.

**Visualization:** Lixian Sun.

**Writing – original draft:** Chao Liu, Haoran Zhang, Weichao Shan.

**Writing – review & editing:** Yanfang Wang, Haoran Zhang.
